# Factors Associated with Maternal Wellbeing at Four Months Post-Partum in Ireland

**DOI:** 10.3390/nu10050609

**Published:** 2018-05-14

**Authors:** Annemarie E. Bennett, John M. Kearney

**Affiliations:** 1 Department of Clinical Medicine, Trinity Centre for Health Sciences, St. James’ Hospital Campus, Dublin 8, Ireland; 2 School of Biological Sciences, Dublin Institute of Technology, Kevin Street, Dublin 8, Ireland; john.kearney@dit.ie

**Keywords:** maternal wellbeing, maternal distress, post-partum distress, breastfeeding support, paternal role, partner support, infant, Ireland

## Abstract

This study aimed to examine factors associated with maternal wellbeing at four months post-partum in the Irish context. Socio-demographic, health behaviour and infant feeding data were collected in pregnancy, at birth and at 17 weeks post-partum. Maternal distress, body image and resilience were measured at 17 weeks post-partum. Binary logistic regression predicted maternal distress and statistical significance was taken at *p* < 0.05. One hundred and seventy-two women were followed-up in pregnancy, at birth and at 17 weeks post-partum. Three in five (61.6%, *n*106) initiated breastfeeding. At 17 weeks post-partum, 23.8% (*n*41) were exclusively or partially breastfeeding and over a third (36.0%, *n*62) of all mothers were at risk of distress. In multivariate analyses, independent predictors of distress included: low maternal resilience (*p* < 0.01, odds ratio (OR): 7.22 (95% confidence interval [CI]: 2.49–20.95)); unsatisfactory partner support (*p* = 0.02, OR: 3.89 (95% CI: 1.20–12.65)); older age (*p* = 0.02, OR: 1.11 (95% CI: 1.02–1.21)); and breastfeeding (*p* = 0.01, OR: 2.89 (95% CI: 1.29–6.47)). Routine assessment of emotional wellbeing and targeted interventions are needed to promote a more healthful transition to motherhood among women in Ireland.

## 1. Introduction

The transition to motherhood is associated with, and most often accompanied by, feelings of maternal joy. However, it is also a time of increased vulnerability and possible distress for women [1], as they process intense and potentially rapid shifts in their emotional state in the period shortly after giving birth [2,3,4]. The postpartum period presents many physiological and psychological challenges to women, such as interrupted sleep, readjustment within the parental relationship, and the need to quickly adapt to new routines and acquire new skills [1]. Challenges such as these can precipitate or amplify maternal distress, particularly if they are accompanied by a lack of social support, low self-esteem or dissatisfaction with the parenting relationship [5].

Maternal distress impairs daily functioning [1,6] and increases the risk of post-partum depression and suboptimal behavioural and emotional development in an infant [6]. Given these possible consequences, it is critical to identify factors associated with maternal distress, in order to understand how it may be attenuated and how the healthy functioning of a mother and her infant may be safeguarded.

Compared to other European countries, Irish women are a singular population when breastfeeding practices are examined [7]. Irish women have the lowest breastfeeding initiation rate in Europe [8,9], rapid declines in rates of exclusive breastfeeding [10], and poor rates of breastfeeding duration beyond the first few weeks post-partum [9]. So extensive and resilient is our formula feeding culture [11], that government investment and policies over the past decade have proved ineffective in increasing breastfeeding rates amongst Irish mothers [7]. As such, it is clear that there are fundamental elements of the breastfeeding environment in Ireland which remain to be understood.

Breastfeeding is a unique component of the post-partum period, where its presence and absence both carry physical, psychological and socio-cultural implications for mothers [12,13]. Given the lack of research in the Irish context, this study aimed to obtain measures of maternal emotional wellbeing and to examine the associations between these measures, if any, with infant feeding outcomes.

## 2. Materials and Methods

Ethical approval for this prospective observational study was obtained from the Coombe Women and Infant’s University Hospital and Dublin Institute of Technology.

Women were recruited by the lead author whilst waiting in community antenatal clinics in the Coombe Women and Infants University Hospital. Eligible women were those who: had a healthy singleton pregnancy; were at least 24 weeks pregnant; were available in hospital for follow-up after labour; and were willing to be contacted in the post-partum period for follow-up via a home visit. Written informed consent was obtained from all participants for all elements of data collection.

A questionnaire was administered in the clinic for pregnant participants to self-complete. This questionnaire collected socio-demographic and health behaviour data, to include: age; nationality; parity; education level; marital status; folic acid supplementation practices; and smoking and alcohol status. The questionnaire also included the validated Tilburg Pregnancy Distress Scale (TPDS) [14].

The TPDS [14] is a 16-item pregnancy-specific scale which measures maternal distress over the 7 days prior to its completion. The overall scale is comprised of two subscales. The negative affect subscale measures distress with respect to confinement, the post-partum period and general health. The partner involvement subscale measures distress with respect to perceived partner involvement during pregnancy. Participants rated on a four-point scale how often they felt as described by each item of the TPDS (i.e., very often; fairly often; now and then; or, rarely or never). Cut-off scores for distress were >17 for the overall scale, >12 for the negative affect subscale and >7 for the partner involvement subscale.

To reduce withdrawal rates and record infant feeding practices, participants were followed-up in hospital after giving birth. The lead author checked hospital records daily to identify those women who had consented to participate in the study and had given birth to a healthy term infant in the previous 24 h. The lead author visited these women on the ward, re-introduced herself and asked them if they were still happy to be contacted at 17 weeks post-partum. The method of infant feeding initiated and the method of infant feeding on discharge from hospital were documented from each mother’s medical record. Standard definitions were used to categorise infant feeding methods [15].

A home visit was arranged during the week in which infants turned 17 weeks of age. During the visit, mothers completed quantitative questions on infant feeding practices and maternal health behaviours. Maternal wellbeing was also assessed using the Mother and Baby Interaction Scale (MABISC) [16], an eight-item Body Shape Questionnaire (BSQ) [17] and the 14-item Resilience Scale [18].

The ten-item MABISC assesses maternal distress and potentially suboptimal mother-infant bonding over the month prior to its completion [16]. Scale items assess worry over bonding, infant caretaking and routine, and separation from the infant. Participants used a five-point scale to rate how often they felt as described by each item (i.e., always; most of the time; occasionally; not often; never). Cut-off scores were ≤7 for no distress, 8–11 for at risk of distress and ≥12 for a high probability of distress. Given the sample size available for multivariate analysis, participants were assigned to the category of no distress (≤7) or the aggregated category of at risk of, or high probability of, distress (≥8).

The eight-item BSQ (version 8b) measures body shape concern over the 7 days prior to its completion [17]. Participants rated on a six-point scale how often they felt as described by each item of the scale (i.e., always; very often; often; sometimes; rarely; never). Cut-off scores for body shape concern were <19 for no concern, 19–25 for mild concern, 26–33 for moderate concern and >33 for marked concern. Given the sample size for analysis, participants were assigned to the category of no concern (<19) or the aggregated category of mild, moderate or marked concern (≥19).

The 14-item Resilience Scale measures resilience, confidence and ability to persevere [18]. Resilience refers to an individual’s ‘emotional stamina’ and their ability to adapt in the face of challenges. Participants rated their agreement with each item on a seven-point scale, where 1 = strongly disagree and 7 = strongly agree. The cut-off scores used in this study were ≤73 for low resilience and ≥74 for moderate to high resilience.

IBM SPSS for Windows, version 22 (IBM, New York, NY, USA) was used for analysis. Normally distributed data were summarised numerically using the mean and standard deviation (SD). Non-normally distributed data were summarised numerically using the median and interquartile range (IQR). To identify factors associated with maternal distress at 17 weeks post-partum, univariate and multivariate analyses were conducted. Associations with continuous variables were determined using Independent Samples *t*-tests, and associations with categorical variables were assessed using 2 × 2 cross-tabulations, where the Chi-squared statistics test assessed statistical significance. Variables which were significantly associated with maternal distress in these univariate analyses were included in multivariate analyses. Binary logistic regression was used to predict maternal distress. The Forced Entry Method was used, whereby all predictor variables were tested in one block to assess their predictive ability whilst controlling for the effects of other predictors in the model. Statistical significance was taken at *p* < 0.05.

## 3. Results

Of the 270 women recruited in pregnancy, 233 were eligible for follow-up in hospital after giving birth (*n*8 infants admitted to intensive care and *n*29 mothers availed of the early discharge service). Of the 233 eligible mothers, 172 (73.8% follow-up rate) consented to follow-up at 17 weeks post-partum (Table 1). Aside from the gestational age of the infant, there were no significant differences between mothers who did and did not consent to follow-up at 17 weeks post-partum.

Over half (56.4%, *n*97) of this sample of 172 women were multiparous. A quarter (25.0%, *n*43) smoked until their pregnancy was confirmed, 7.6% (*n*13) smoked all throughout pregnancy, and 22.1% (*n*38) consumed alcohol during their pregnancy. Only a third (33.1%, *n*57) supplemented with folic acid in line with recommendations [19].

### 3.1. Milk-Feeding Practices

Three in five (61.6%, *n*106) mothers initiated breastfeeding upon the birth of their infant. The median length of stay in hospital was 48.2 (37.8) h, and by discharge, the proportion of women exclusively breastfeeding had decreased to 37.2% (*n*64). A further 15.7% (*n*27) were combination feeding, and almost half (47.1%, *n*81) were solely formula feeding upon discharge.

Of those who initiated breastfeeding (*n*106), the majority (86.8%, *n*92) reported that their partner was completely supportive of their decision to breastfeed. One in eight (12.3%, *n*13) reported that their partner was mostly supportive and one mother (0.9%) reported that her partner was not supportive of her decision to breastfeed. Less than half (44.3%, *n*47) reported putting specific breastfeeding supports in place prior to the birth of their infant (e.g., making contact with local breastfeeding support groups).

By 17 weeks post-partum, the proportion of mothers exclusively breastfeeding had decreased to 15.1% (*n*26), with a further 8.7% (*n*15) combination feeding their infant. Over three-quarters (76.2%, *n*131) were solely formula-feeding their infant at this time.

### 3.2. Maternal Wellbeing in Pregnancy and at 17 Weeks Post-Partum

A quarter (25.0%, *n*43) of the 172 women followed-up in the post-partum period had met or exceeded the cut-off [14] for distress in pregnancy (Table 2). Over a quarter (27.9%, *n*48) were distressed according to the negative affect subscale of the TPDS, and one in ten (9.9%, *n*17) were distressed on the partner involvement subscale.

At 17 weeks post-partum, almost two-thirds (64.0%, *n*110) were coping well and not distressed (Table 2) according to the scoring of the MABISC [16]. Over a quarter (27.9%, *n*48) were at risk of distress and one in twelve (8.1%, *n*14) met cut-offs for a high probability of distress. Of note, different scales were used to determine the prevalence of distress at each time point.

Over half (52.9%, *n*91) of mothers had no body shape concern at 17 weeks post-partum. A quarter (26.2%, *n*45) had mild body shape concern and 14.0% (*n*24) and 7.0% (*n*12) had moderate and marked body shape concern, respectively. At 17 weeks post-partum, 13.3% (*n*23) of mothers had low levels of resilience and 86.6% (*n*149) had high levels of resilience.

### 3.3. Factors Associated with Maternal Wellbeing at 17 Weeks Post-Partum

The characteristics of women who experienced no distress at 17 weeks post-partum (64.0%, *n*110) were compared with those of mothers experiencing some degree (high probability/at risk) of distress at this time (36.0%, *n*62) (Table 3). From these univariate analyses, distress at 17 weeks post-partum was more likely if a mother was: older (*p* < 0.01); multiparous (*p* = 0.02); breastfeeding (*p* < 0.01); or distressed by her partner’s involvement (or lack thereof) in pregnancy (*p* = 0.02). Distress was also more likely if a mother had low resilience (*p* < 0.01).

Multivariate analyses were conducted to examine the characteristics independently associated with distress at 17 weeks post-partum (Table 4). All variables entered into the model were categorical, except for age, which was a continuous variable. As shown in the statistically significant adjusted model (χ^2^ (6, *n*172) = 43.15, *p* < 0.01), four of the six independent variables included made a statistically significant contribution to the model. The strongest predictor of distress at 17 weeks post-partum was low resilience. Mothers with low resilience scores were over seven times more likely (Table 4) to be at risk of distress when compared with mothers with high resilience scores. Mothers were also significantly more likely to feel distressed at 17 weeks post-partum if they were older, breastfeeding at this time or had been distressed by partner involvement (or lack thereof) in pregnancy (Table 4).

## 4. Discussion

Giving birth and caring for a new infant marks an important transition from one stage of a woman’s life to another. Although this transition is often joyous, it is also a particularly challenging chapter of parenting which involves readjustment of roles and rapid acquisition of new skills. The unpredictability and upheaval associated with this time can, in turn, increase the risk of emotional distress amongst women. In this study, several factors were independently associated with post-partum distress (as measured by the MABISC), to include: low maternal resilience; suboptimal partner support; older age; and, breastfeeding. In particular, the association between breastfeeding and distress was somewhat unexpected, where breastfeeding women were almost 3 times more likely to experience distress at 17 weeks post-partum when compared with non-breastfeeding women.

Breastfeeding is often regarded as a challenging demand of motherhood [4], particularly in Ireland, which has a long-reigning formula feeding culture [7,11,20] and widely reported inadequate breastfeeding support [21,22,23]. Women who breastfeed at 17 weeks post-partum are in the minority, with only one in five Irish women breastfeeding, exclusively or otherwise, at this time. If suboptimal breastfeeding rates [8] are to be subverted, a more comprehensive understanding of the feeding experiences of women who persevere with breastfeeding beyond the first few weeks post-partum must be obtained.

Meedya and colleagues [24] identified three factors which lend themselves to a breastfeeding experience in which maternal distress is less likely to occur, namely: a positive antenatal intention to breastfeed; strong social support; and high maternal resilience.

A positive antenatal intention to breastfeed has been strongly and consistently associated with breastfeeding initiation and duration [9,24]. Pregnancy is an important opportunity to prepare for breastfeeding, but less than half of the women who intended to breastfeed in this study put supports in place to increase the likelihood of a positive breastfeeding experience. Given that breastfeeding impacts on so many aspects of a woman’s day-to-day life (i.e., her physical health, mental health, and social and cultural activities), women must be as prepared as possible for the challenges that breastfeeding may pose to these aspects of life after pregnancy [12,13]. Such preparation is needed to minimise any discrepancies between a woman’s expectations of breastfeeding and the reality of breastfeeding [25].

When significant discrepancies between expectations and reality arise, disillusionment with breastfeeding can manifest, increasing the likelihood of maternal distress [26]. However, pregnancy presents an ample opportunity for health services to capitalise upon positive intentions to breastfeed. During their frequent points of contact with pregnant women, health professionals should help women to develop not only a positive intention to breastfeed—but an informed positive intention to breastfeed. Educating pregnant women on breastfeeding can empower them to identify suitable breastfeeding supports that they can independently access in a timely manner to help them to acquire the skill of breastfeeding upon their infant’s arrival [12,27,28].

Supporting women to support themselves is especially important in light of the recent decision to cease all funding for, and activities related to, the Baby Friendly Health Initiative (BFHI) in Ireland [29]. The BFHI aims to introduce evidence-based and sustainable practices which promote and protect breastfeeding in maternity hospitals [30]. However, despite being in place for almost two decades in Ireland, the progress made by the National Committee of the BFHI was deemed insufficient by the relevant government agency in 2017, resulting in the controversial withdrawal of all funding for BFHI activities [29]. At its best, approximately one in three infants in Ireland were born in BFHI hospitals [30], so a deficit in breastfeeding supports in the immediate aftermath of birth has long existed here, and unfortunately has now only deepened with the absence of any formal implementation of an evidence-based breastfeeding support system.

Given the widespread lack of standardised support at hospital-level [29], social support is a particularly important element of a positive breastfeeding experience [9,21,24,31]. Specifically, the support from a woman’s partner impacts on her feeding experience [24], and it is positive that in this study, most partners supported the decision to breastfeed. However, whilst partners may support this decision based on a superficial understanding of the immense value of breastfeeding, they often do not have an informed understanding of the process of breastfeeding and of the unique support it requires [21]. Several studies have reported that educating fathers on the fundamentals of breastfeeding results in improvements in rates of breastfeeding initiation and duration, and fewer technical difficulties with feeding [24,32,33,34,35]. Breastfeeding women have also reported that teaching fathers to provide specific types of practical (such as help with cooking, housework and caring for other children) and emotional support (such as praising their breastfeeding efforts and defending them from suggestions to formula-feed) enhances their breastfeeding experience and their ability to cope with breastfeeding challenges, should they arise [1]. Fathers can provide a continuity of care to a mother which no health professional can offer, and as such, educating fathers on how to best participate in the breastfeeding process can serve as a valuable means of promoting a more supportive and less isolating breastfeeding experience for a woman.

In addition to external support from health professionals and partners, it is also important that women view themselves as a source of support, in that they possess the resilience and equanimity to manage the responsibility of being the sole food provider for an infant [13]. Low maternal resilience was a separate independent predictor of maternal distress in this study, but it is also a factor which can impact on the breastfeeding experience.

Research has demonstrated the effectiveness of using mindfulness and cognitive behavioural therapy techniques to enhance maternal resilience and elicit clinically significant reductions in distress, depression and anxiety [6,36,37,38]. For example, mindfulness-based cognitive behavioural therapy has been shown to help pregnant women to foster acceptance, manage negative thoughts and deal with obstacles [38]. Helping pregnant women to strengthen their mental capacity to cope with the many elements that comprise the transition to motherhood could be a valuable means of preventing or attenuating distress which may arise during this time.

Perseverance with breastfeeding, despite its associated physical and emotional challenges, has been well-documented [4,12,26]. Although it may seem counterintuitive for a woman to persevere with an activity which may be a source of distress, it is likely that the commitment to, and value placed upon, breastfeeding by some women is so great that distress related to an aspect or aspects of breastfeeding is made tolerable or at least reduced. This commitment to breastfeeding may enable some women to continue to breastfeed despite any shortcomings in their support network or resilience [3,39]. However, while it is important to protect breastfeeding, equal emphasis should be placed on protecting the emotional wellbeing of women as they meet the demands of breastfeeding.

Finally, regardless of the infant feeding decision made, it is important that all women are appropriately supported throughout their transition to motherhood. Women with low resilience levels, suboptimal partner support, or who were older, were also more likely to feel distressed in this study. Many of the factors discussed here, support breastfeeding secondary to supporting a woman’s overall emotional wellbeing [36,37,40]. A healthy emotional state underpins a woman’s ability to carry out any element of her mothering role, breastfeeding or otherwise; breastfeeding is one element of motherhood, and just as it requires empowerment, education, resilience and support, so too do many other parts of this role. Routine assessment of emotional wellbeing and targeted interventions are needed to promote a more healthful transition to motherhood among all women who choose to have children.

Before drawing conclusions, the study strengths and limitations must be considered. The data presented were collected as part of a longitudinal observational study conducted in County Dublin and its surrounding counties. Strengths of the study include the lack of inter-observer variation and the use of validated instruments to measure different aspects of maternal wellbeing. The MABISC has been shown to have satisfactory internal consistency and convergent validity with the more widely used Edinburgh Post-partum Depression Scale (EPDS) and Post-partum Bonding Questionnaire (PBQ) [16]. Although the EPDS and PBQ are more helpful for the detection of the most serious maternal distress and rejection problems, the MABISC was suitable for use in this study due to its brevity and the inoffensive phrasing of items. However, when interpreting the findings, it is important to note that the observational study design precludes causal inferences, involves only women of Irish or British nationality, and is not nationally representative.

The relationship between breastfeeding and many sociodemographic and health behaviour characteristics has been examined in the Irish context [7,8,11,23,41]. Bearing the study limitations in mind, this is one of the first studies to provide insights into the relationship between emotional wellbeing and infant feeding practices amongst a cohort of mothers in Ireland.

## 5. Conclusions

Adequate physical, emotional and practical preparation for breastfeeding is paramount to its successful initiation and maintenance. Pregnant women planning to breastfeed should be empowered with practical and evidence-based information on feeding techniques and positioning, in addition to information on troubleshooting common breastfeeding challenges. All women should also be supported to attain and maintain a healthy body and frame of mind prior to giving birth. If this preparation among women is further bolstered by a well-prepared maternal support network, women can approach motherhood, whether it is for the first or subsequent time, in an informed manner which safeguards their wellbeing and that of their infant.

## Figures and Tables

**Table 1:** Socio-demographic and health behaviour characteristics of 172 participating women.

	Mean ± SD	
Age on Delivery (years)	32.0 ± 4.8	
	*n*	%
Nationality		
Irish	169	98.3
British	3	1.7
Highest education level		
Third level (university)	111	64.6
Vocational qualification	19	11
Second level (secondary school)	42	24.4
Marital status		
Married or cohabiting	151	87.8
Single	21	12.2
Health insurance		
Semi-private	57	33.1
Public	115	66.9
Planned pregnancy		
Yes, planned	127	73.8
No, unplanned	45	26.2
Body mass index		
Healthy	83	48.3
Overweight	68	39.5
Obese	21	12.2

SD: Standard deviation.

**Table 2:** Distress in pregnancy and at 17 weeks post-partum in a sample of 172 women giving birth in Ireland.

	*n*	%
Distress in pregnancy		
Tilburg Pregnancy Distress Scale (TPDS)		
Significant distress	43	25
No significant distress	129	75
Negative affect subscale of TPDS		
Significant distress	48	27.9
No significant distress	124	72.1
Partner involvement subscale of TPDS		
Significant distress	17	9.9
No significant distress	155	90.1
Distress at 17 weeks post-partum		
Mother and Baby Interaction scale		
High probability of distress	14	8.1
At risk of distress	48	27.9
No distress	110	64

**Table 3:** Comparison of maternal characteristics associated with distress at 4 months post-partum.

	Significant Distress at 4 Months Post-Partum (*n*62)		No Significant Distress at 4 Months Post-Partum (*n*110)		*p*-Value *
Maternal age at delivery (years)	*n*	Mean ± SD	*n*	Mean ± SD	**<0.01 ‡**
	62	33.8 ± 4.2	110	31.0 ± 4.9	
	*n*	%	*n*	%	
Maternal education					0.14 †
Third level education	45	72.6	66	60	
No third level education	17	27.4	44	40	
Unplanned pregnancy	18	29	27	24.5	0.64 †
No partner involved in pregnancy	3	4.8	2	1.8	0.26 †
Parity					**0.02 †**
Nulliparous	20	32.3	55	50	
Multiparous	42	67.7	55	50	
Smoked in all three trimesters	4	6.5	9	8.2	0.91 †
Consumed alcohol in pregnancy	15	24.2	23	20.9	0.76 †
Maternal body mass index					0.62 †
≤24.9 kg/m^2^	32	51.6	51	46.4	
≥25.0 kg/m^2^	30	48.4	59	53.6	
Significantly distressed on TPDS					
Overall scale	20	37.7	23	41.8	0.14 †
Negative affect subscale	22	41.5	26	47.3	0.13 †
Partner involvement subscale	11	20.7	6	10.9	**0.02 †**
First milk					0.48 †
Breast milk	43	69.4	69	62.7	
Formula milk	19	30.6	41	37.3	
Breastfeeding at 4 months post-partum	24	38.7	17	15.5	**<0.01 †**
Has supports in place to breastfeed	16	41	31	46.3	0.75 †
Post-partum body shape concern					0.46 †
No concern	32	51.6	49	44.5	
Mild, moderate or marked concern	30	48.4	61	55.5	
Post-partum resilience category					**<0.01 †**
High resilience	46	74.2	103	93.6	
Low resilience	16	25.8	7	6.4	
Not weaned before 17 weeks of age	52	89.7	87	83.7	0.42 †

SD: Standard deviation; TPDS: Tilburg Pregnancy Distress Scale; * *p*-value of <0.05 was significant; ‡ Association between normally distributed continuous data assessed with an Independent Samples *t*-test; † Association between categorical variables assessed using a chi-squared test with Yates’ Continuity Correction for 2 × 2 contingency tables.

**Table 4:** Binary logistic regression model examining factors associated with distress at 17 weeks post-partum in a sample of 172 mothers who gave birth in Ireland.

	*n*	OR	95% CI	*p*-Value *
Third level education				
Yes	111	1.4	0.63–3.12	0.41
No	61	1	Ref.	
Parity				
Primiparous	75	1	Ref.	0.24
Multiparous	97	1.6	0.74–3.47	
Distressed by partner involvement in pregnancy †				
Yes	17	3.89	1.20–12.65	**0.02**
No	155	1	Ref.	
Resilience level at 17 weeks post-partum				
High	149	1	Ref.	**<0.01**
Low	23	7.22	2.49–20.95	
Breastfeeding at 17 weeks post-partum				
Yes	41	2.89	1.29–6.47	**0.01**
No	131	1	Ref.	
Maternal age	172	1.11	1.02–1.21	**0.02**
Model summary				
R^2^ = 0.16, Cox & Snell R Square = 22.2, Nagelkerke R Square = 30.4, 72.7% predictive of variance				

* p-value significant at <0.05; OR: odds ratio; CI: confidence interval; † Distress measured by the Tilburg Pregnancy Distress Scale [14].
